# Machine Learning Model for Predicting Sertraline-like Activities and Its Impact on Cancer Chemosensitization

**DOI:** 10.1021/acschemneuro.5c00165

**Published:** 2025-06-23

**Authors:** Jin-Yu Xia, Ze-Yu Sun, Ying Xue, Ying-Qian Zhang, Zhi-Wei Feng, Yu-Long Li

**Affiliations:** 1 School of Civil Engineering, College of Chemistry and Environmental Engineering, 74601 Sichuan University of Science and Engineering, Zigong 643000, P. R. China; 2 Department of Pharmaceutical Sciences and Computational Chemical Genomics Screening Center, School of Pharmacy, 6614 University of Pittsburgh, Pittsburgh, Pennsylvania 15261, United States; 3 Faculty of Pharmaceutical Sciences, Shenzhen University of Advanced Technology, Shenzhen 518107, P. R. China

**Keywords:** machine learning, SSRI activity prediction, escitalopram analogs, drug discovery, feature engineering, predictive model

## Abstract

Selective serotonin reuptake inhibitors (SSRIs) like sertraline are crucial in treating depression and anxiety disorders, and studies indicate their potential as chemosensitizers in cancer therapy. This research develops a machine-learning predictive model to identify novel compounds with sertraline-like antidepressant activity. We constructed and validated a customized machine-learning model to predict SSRI activity in new compounds. By applying feature engineering to the chemical structures and bioactivity data of sertraline and its analogs, we trained multiple machine-learning algorithms. Through extensive comparative analysis, we found that the support vector machine (SVM) model demonstrated exceptional performance, achieving an accuracy rate of up to 93%. By further optimizing and integrating the SVM model, we successfully enhanced its accuracy, reaching an impressive 95% capability in predicting more active SSRI compounds. This study successfully developed a targeted, rapid, and efficient machine learning model capable of accurately predicting SSRI activity. The model serves as a valuable tool for rapidly screening novel SSRI drug candidates with superior activity, bringing immense value to the field of drug development.

## Graphical abstract

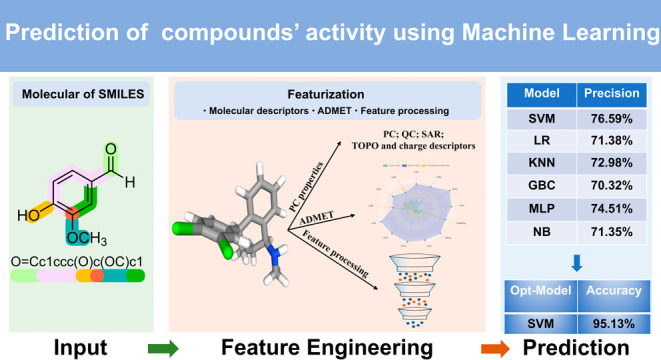

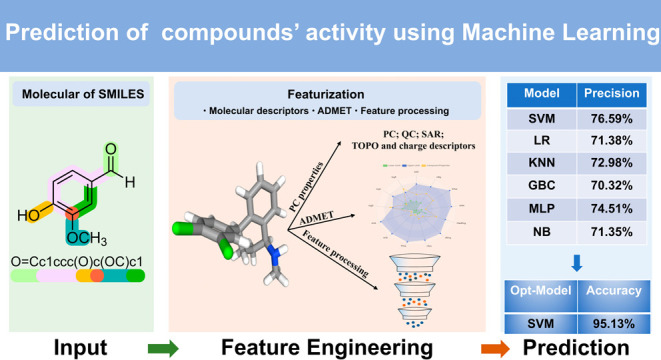

## 1 Introduction

Gastric cancer poses a significant health challenge globally, ranking among the leading causes of cancer-related mortality. According to statistical reports, China bears a substantial burden, accounting for 44.0% and 48.6% of the world’s incidence and mortality rates, respectively.^1^ In the treatment of gastric cancer, chemotherapy remains a crucial option for patients with advanced-stage disease.^2^ However, the emergence of resistance and the formidable obstacle of multidrug resistance have regrettably diminished the efficacy of chemotherapy. A concerning phenomenon observed in gastric cancer cells is their ability to exhibit resistance not only to chemotherapeutic agents but also to targeted drugs, ultimately leading to suboptimal therapeutic outcomes.^3^

According to relevant research, cancer cells develop drug resistance through various mechanisms,^4,5^ including genetic mutations and changes in signaling pathways. Despite the complex and diverse nature of cancer cell drug resistance mechanisms, they all involve the process of cell apoptosis. By inducing cancer cell apoptosis with adjuvant drugs, such as chemosensitizers,^6,7^ we can enhance the effectiveness of chemotherapy drugs. This strategy aims to trigger the apoptotic pathway in cancer cells, thereby eliminating drug-resistant cells. Through the induction of apoptosis, we can increase the cytotoxicity of chemotherapy drugs on cancer cells, improving treatment outcomes. Therefore, developing adjuvant drugs that can induce cancer cell apoptosis, such as chemosensitizers, becomes a crucial strategy for enhancing gastric cancer treatment efficacy. By employing this approach, we can overcome cancer cell drug resistance and improve treatment success rates.^8^

One promising avenue involves the repurposing of existing drugs in strategic combination with chemotherapy agents,^4,9^ aiming to bolster their effects and improve overall treatment efficacy. For instance, sildenafil, initially introduced as a therapeutic for erectile dysfunction, has revealed an intriguing capacity to sensitize cancer cells to the effects of chemotherapy and radiotherapy, thereby enhancing the overall effectiveness of these treatments.^10,11^ Statins, widely recognized for their cholesterol-lowering properties, have also emerged as potential chemosensitizers, augmenting the impact of chemotherapy.^12,13^ Prior research has indicated that selective serotonin reuptake inhibitors (SSRIs), a class of antidepressants, possess anticancer attributes.^14,15^ Among them, sertraline has demonstrated notable potential in sensitizing resistant gastric cancer cells to the effects of chemotherapy drugs.^16^ Studies suggest that sertraline exerts its influence on resistant gastric cancer cells by inducing apoptosis and cell cycle arrest, providing a promising direction for the development of novel chemical sensitizers.^16^

Sertraline, as a chemosensitizing agent, primarily exerts its activity by enhancing the antitumor effects of chemotherapy drugs through a multitarget mechanism. Studies have shown that when sertraline is combined with various chemotherapy agents (such as doxorubicin, vincristine, and erlotinib), it can reduce drug efflux by inhibiting ATP-binding cassette (ABC) transporters, thereby increasing intracellular chemotherapy drug concentrations.^17^ Additionally, sertraline enhances drug sensitivity by inducing autophagy and apoptosis through modulation of the mTOR/Akt signaling pathway.^18^ Its ability to inhibit TCTP expression can restore p53 function, further promoting tumor cell apoptosis and reversing chemoresistance.^19,20^ Relevant experimental data demonstrate that sertraline combination therapies significantly reduce tumor volume and recurrence rates in various models, including breast cancer, lung cancer, and melanoma.^21,22^ These mechanisms involve the regulation of Bcl-2 family proteins, activation of caspase-3/7, and alterations in calcium ion homeostasis,^23,24^ highlighting its potential as a multimodal sensitizing agent.

Despite sertraline’s demonstrated multitarget potential in reversing gastric cancer drug resistance, its structure–activity relationship (SAR) studies for antigastric cancer activity remain in the preliminary stages. Molecular mechanisms indicate that their pharmacological effects are closely associated with specific functional groups: Mu et al.^25^ confirmed through structural modification experiments that the hydrogen atom (H) on the secondary amine group of sertraline is an essential moiety for activity Figure 1. Replacing this hydrogen with a methyl group completely abolished the drug’s antigastric cancer activity. Bin Kanner et al.^26^ revealed through molecular docking that the hydrophobicity of its dichlorophenyl group enhances binding affinity to ABC transporters, underscoring the critical role of hydrophobic moieties in overcoming drug resistance.

**Figure 1:**
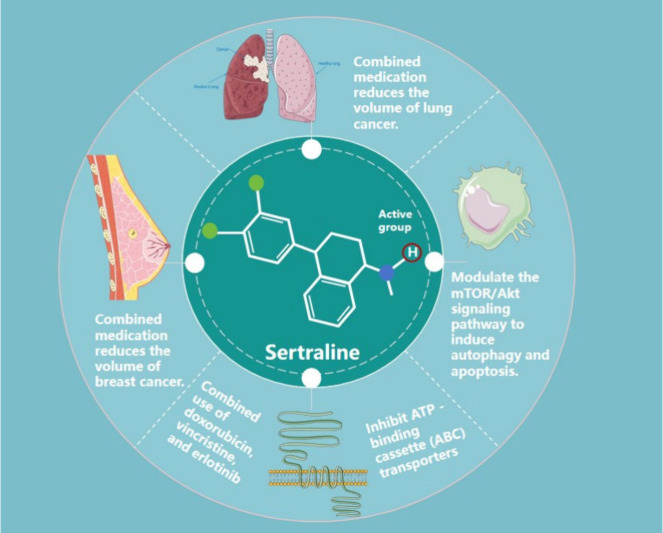
Key active groups of sertraline and their anticancer action pathways.

Current research focuses solely on single-point validation of individual functional groups, lacking systematic SAR analysis, which limits the structural optimization and development of highly efficient sensitizing agents. This gap provides an entry point for machine learning intervention. By integration of fragmented data to construct SAR prediction models, machine learning can accelerate the activity screening and mechanism elucidation of sertraline derivatives. Machine learning (ML) has revolutionized the landscape of drug discovery, profoundly impacting traditional research methodologies.^27^ By harnessing the power of ML to predict the activity of sertraline analogs, we can significantly expedite the drug repurposing and development process.

This study aims to bridge this gap by developing a sophisticated machine learning framework designed to predict the activity of sertraline analogs in the context of combating drug-resistant gastric cancer. By leveraging established structure–activity relationships and delving into the biological mechanisms that underpin drug resistance, our ML model aims to identify prospective drug candidates that warrant further experimental validation. The successful development and deployment of this ML model hold the promise of not only accelerating the discovery of innovative chemical sensitizers but also contributing to a deeper understanding of the intricate molecular interactions that occur between sertraline derivatives and drug-resistant gastric cancer cells. This cutting-edge approach has the potential to reshape the therapeutic landscape for gastric cancer, offering new hope to patients confronting the formidable challenge of drug resistance.

## 2 Materials and Methods

### 2.1 Data Set Collection

To further our investigation, we meticulously collated structural and activity data for a diverse set of 268 sertraline-related derivatives from previously published literature,^27^ patents, and the Zinc database. (The Zinc compound data can be referenced from the “data.csv” file located at the following Web site: https://github.com/xiaxiazainuli/A-Machine-Learning-Model-for-Predicting-Sertraline-like-Activities.git.) The sources of data and the criteria for division are classified in detail as follows.

The first study^16^ synthesized 32 new compounds and tested their combinatorial effect with paroxetine on cisplatin-resistant gastric cancer cell lines to evaluate their antiproliferative potential against cancer cell growth. The results revealed that eight derivatives (5t, 6a, 6a′, 6b, 6b′, 6c, 6c′, and 6d) exhibited superior antiproliferative activity compared to paroxetine. Based on sertraline as a reference, compounds with lower activity than sertraline are classified as low-activity compounds and are scored as 0. Table 1 determines the IC_50_ values of the compounds, highlighting their potency.

**Table 1:** IC_50_ Values of the Compounds with Superior Activity, Serial Numbers 1–33

entry	compound	cis/trans	IC_50_ (μM)
1	sertraline	cis	18.73 ± 0.46
2	5t	trans	17.05 ± 0.82
3	6a	trans	10.96 ± 0.51
4	6a′	cis	10.69 ± 0.41
5	6b	trans	6.28 ± 0.50
6	6b′	cis	6.30 ± 2.46
7	6c	trans	11.10 ± 0.65
8	6c′	cis	18.90 ± 0.51
9	6d	trans	5.20 ± 0.36

This patent^30^ invention synthesized paroxetine-like compounds and assessed their antidepressant activity through mouse experiments. Researchers prepared a mixture of the compounds with paroxetine and a 0.5% sodium carboxymethyl cellulose solution to administer drugs to mice and observe their immobility time under various conditions. Table 2 presents the compounds that exhibited superior activity compared to that of paroxetine. The classification method is the same as above. The classification method is the same as described above, using sertraline as the reference for categorization.

**Table 2:** Biological Activity of the Synthesized Compounds with Serial Numbers 34–147 Was Assessed in Mouse Tail Suspension and Forced Swimming Experiments with Paroxetine as the Control Group

sample name	tail suspension experiment (immobilization time in seconds)	forced swimming experiment (immobilization time in seconds)
solvent control group	95.4(±38.8)	123.6(±52.3)
sertraline	48.3(±18.2)	58.7(±42.7)
2	41.1(±13.4)	49.4(±13.5)
3	40.7(±11.7)	47.4(±15.2)
4	37.1(±12.3)	43.6(±20.6)
5	39.2(±13.0)	46.0(±32.2)
9	37.4(±15.2)	51.1(±34.5)
13	37.2(±15.8)	51.4(±34.5)
19	39.2(±7.8)	50.5(±20.4)
20	37.6(±12.3)	44.1(±30.8)
30	40.1(±13.4)	48.1(±33.0)
31	36.5(±12.5)	43.4(±30.2)
39	40.6(±13.3)	49.1(±33.4)
53	35.0(±12.1)	41.1(±28.2)
54	39.5(±13.2)	46.4(±22.2)
55	38.0(±7.0)	49.1(±26.2)
57	36.0(±12.1)	42.5(±30.0)
59	41.6(±14.2)	48.1(±24.4)
67	38.0(±12.5)	44.5(±21.1)
76	39.2(±9.1)	49.4(±17.2)
77	38.4(±13.1)	45.4(±21.6)
78	39.3(±8.0)	50.7(±22.1)
81	35.4(±12.1)	42.0(±29.2)
93	40.0(±10.8)	50.1(±11.1)
97	40.4(±10.4)	50.1(±10.2)
101	39.7(±13.3)	47.4(±22.4)

This paper^29^ designed new derivatives through scaffold hopping based on paroxetine. By determining and comparing the IC_50_ values of the synthesized compounds with paroxetine (Table 3), derivatives with higher activity than sertraline are classified as class 1, while those with lower activity are classified as class 0.

**Table 3:** IC_50_ Values of the Newly Synthesized Compounds with Serial Numbers 148–171 in Comparison to Paroxetine

compound	IC_50_ (μg/mL)	compound	IC_50_ (μg/mL)
sertraline	8	D10	16
cis-sertraline	8	D11	32
trans-sertraline	8	D12	8
D1	8	D13	4
D2	32	D14	1
D3	16	D15	2
D4	8	D16	0.5
D5	8	D17	2
D6	4	D18	4
D7	2	D19	64
D8	32	D20	32
D9	16	D21	16

This study^28^ aimed to design and synthesize a series of sertraline derivatives by introducing polar groups to the fused tetrahydronaphthalene ring to reduce the distribution volume. This strategy was employed to expedite the time to the maximum plasma concentration and enhance the rate of increase in central 5-hydroxytryptamine (5-HT) levels. Table 4 lists the IC_50_ values of the compounds compared to those of sertraline, and the compounds are classified by comparison, with the classification method still using sertraline as the reference.

**Table 4:** IC_50_ Values of the Compounds with Serial Numbers 172–206 in Comparison to Paroxetine

compound	h-SRI (IC_50_, nM)	compound	h-SRI (IC_50_, nM)
sertraline	3	t	290
a	31	u	240
b	8	v	25
c	31	w	5
d	140	x	70
e	5	y	5
f	90	z	4
g	30	1y	6
h	3	2y	1
i	2	3y	56
g	2	4y	7
k	3	5y	54
l	9	6y	13
m	10	7y	25
n	3	8y	7
o	10	9y	60
p	2	10y	>1000
q	6	11y	24
r	340	12y	65
s	1		

This extensive data set has been categorized into two distinct classes based on a comparative analysis of their potency levels relative to sertraline. Compounds that exhibited higher pharmacological efficacy compared to sertraline were designated as class 1, while those displaying lower potency were assigned to class 0. For a detailed breakdown of this classification, refer to Table 5.

**Table 5:** To Compile a Data Set Related to Esculetin Derivatives and Organize It into CSV Format for Training Purposes

index	activity	SMILES
0	0	ClC1=CC=C([C@H]2C3=CC=CC=C3[C@@H](N(C4CC4)CO)CC2)C=C1Cl
1	0	ClC1=CC=C([C@H]2C3=CC=CC=C3[C@@H](N(C4CC4)COC)CC2)C=C1Cl
...	0	ClC1=CC=C([C@H]2C3=CC=CC=C3[C@@H](N(C4CC4)COCC)CC2)C=C1Cl
268	1	ClC1=CC=C([C@H]2C3=CC=CC=C3[C@@H](N(C4CC4)COCCC)CC2)C=C1Cl

We leverage the powerful Python library RDKit, Figure 2, to parse SMILES strings,^31^ offering efficient and robust computational performance. With RDKit, we can extract a wealth of physicochemical properties from the SMILES formulas (Table 6), including molecular weight, lipophilicity, and polar surface area, among others. These critical parameters lay the foundation for subsequent analysis and modeling endeavors.

**Figure 2:**
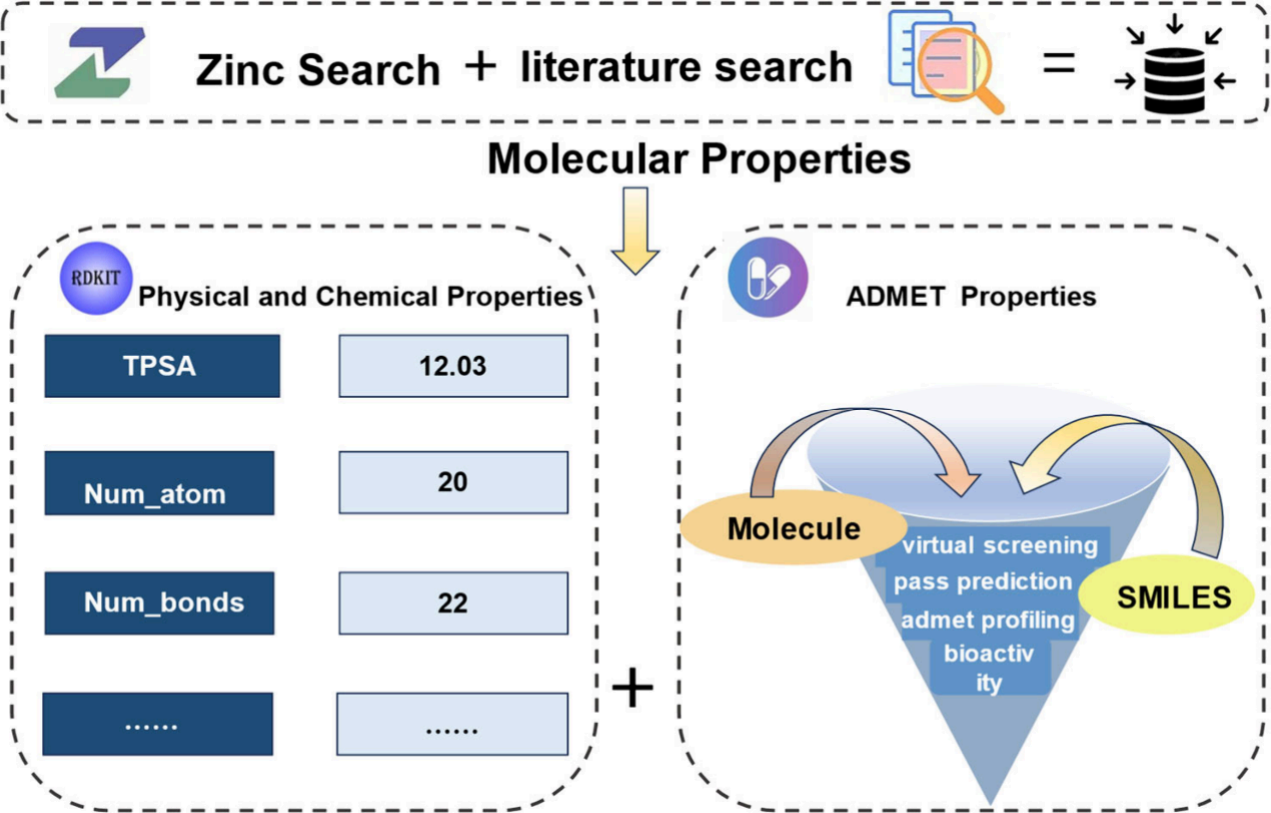
The data set obtains physicochemical properties using RDKit and obtains ADMET and related data through the ADMET 3.0 testing platform.

**Table 6:** Physicochemical Properties Extracted through RDKit Encompass a Comprehensive Set of Molecular Descriptors for Each Compound

Index	Num-atoms	Balaban_j	Wienerindex	LogP
0	20	2.046238	306.236	5.1796
1	21	2.045651	320.263	5.5218
...	...	...	...	...
266	20	2.046238	306.236	5.1796
267	20	2.046238	306.236	5.1796

After acquiring the physicochemical property data, we employ the advanced ADMET 3.0 (https://admetlab3.scbdd.com/) model to predict crucial characteristics of the compounds,^32^ including drug metabolism, absorption, distribution, excretion, and toxicity (ADMET). ADMET 3.0 is a potent tool that integrates quantum mechanics and molecular mechanics techniques, offering valuable insights into these intricate predictions. This approach enables us to better evaluate the potential efficacy and safety of drug candidates.

### 2.2 Feature Engineering

By performing the aforementioned feature extraction Figure 3, we obtained 88 physicochemical features and subsequently embarked on a series of preprocessing steps for the feature data.^33^ First, we analyzed the feature data by computing the variance of each feature to understand the magnitude of variation within the data. We established a threshold to exclude features with high variance, excessive noise, and instability. Features with exceedingly high variance can lead to issues such as model overfitting, challenges in data normalization, and computational inefficiency. Specifically, we opted to use the upper quartile to set the threshold for variance, which was calculated at 0.028. This process resulted in the retention of 12 features. Moving forward, we enhanced the quality of the data by addressing outliers, as they can bias the model, hinder its ability to capture true trends and relationships in the data, and diminish model performance. Here, we employed the robust isolation forest^34^ algorithm to further scrutinize and eradicate outliers from the data set. Due to the small number of detected outliers, we opted to fill the missing values with the mean, preserving sample size and information. Subsequently, we analyzed the contribution of the feature variables to the target variable, which is a binary classification problem, with one class representing higher activity (labeled as 1) and the other class representing lower activity (labeled as 0). To optimize the feature selection process, we employed a recursive elimination method. The recursive elimination method involves iteratively creating models, retaining the best- and worst-performing features in each iteration. This selection process culminated in the retention of 12 features with their respective collinearity levels for the target variable. Upon identifying the highly correlated features, we proceeded to address the issue of collinearity among the features, as it can lead to inaccurate coefficient estimates, decreased explanatory power, and challenges in feature selection.^35^ To tackle this, we employed a feature combination approach,^36^ introducing nonlinear relationships to better fit the model and enhance its expressive capacity. Specifically, we opted to use the ratio of collinear features to generate a new set of features. The new feature set was then utilized for model fitting.

**Figure 3:**
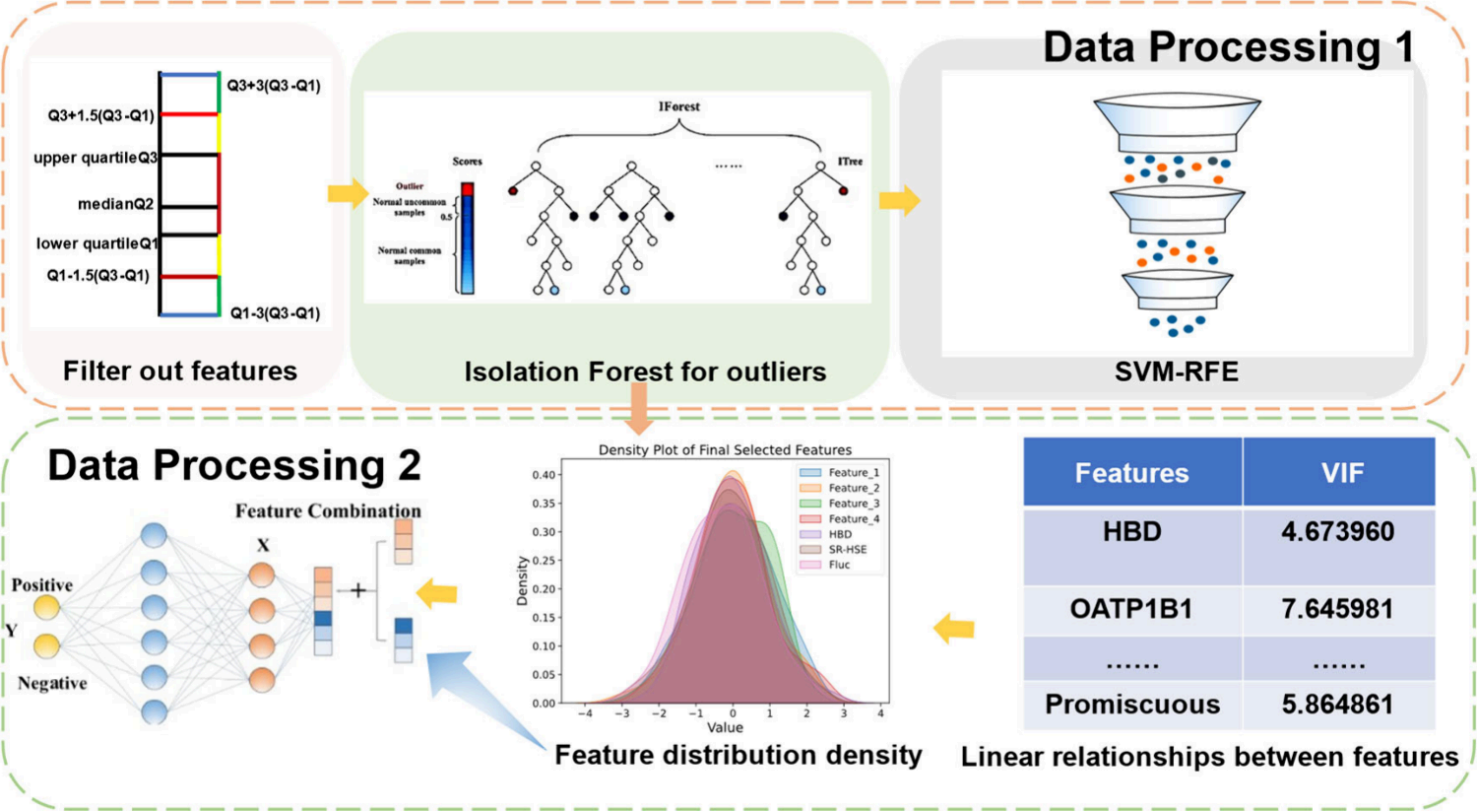
We employed an upper quartile range filter for features with high variance, utilized isolation forest for outlier detection, and selected the best features using recursive feature elimination. VIF values were calculated and visualized to address the multicollinearity. Finally, principal component analysis (PCA) was applied to handle multicollinearity and optimize model input features.

### 2.3 Model Selection

Upon completion of the feature engineering phase, we entered the critical stage of model selection and optimization. In this phase, we deployed an array of advanced machine learning algorithms that are renowned for their prowess in binary classification tasks. Six robust algorithms were chosen: logistic regression (LR), gradient boosting (GB), naive Bayes (NB), support vector machine (SVM), K-nearest neighbors (KNN), and multilayer perceptron (MLP) Figure 4. Given the binary classification problem at hand (predicting whether a compound exhibits activity), we focused on training and optimizing these six algorithms to attain optimal predictive performance.

**Figure 4:**
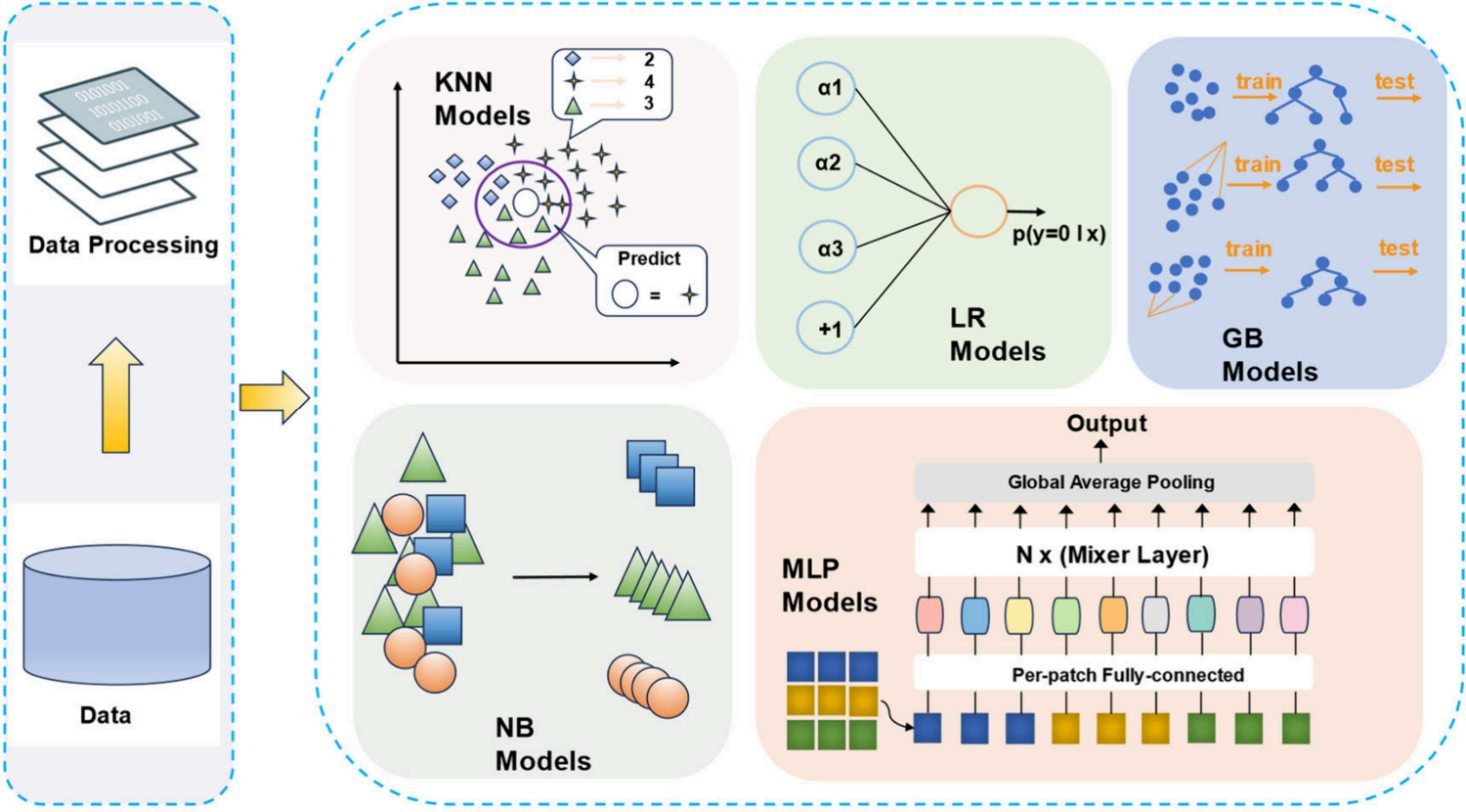
Five machine learning models related to classification: K-nearest neighbors (KNN), naive Bayes (NB), multilayer perceptron (MLP), and logistic regression (LR), as well as gradient boosting (GB).

The hyperparameters of each model were meticulously tuned to ensure their ability to accurately capture intricate patterns and relationships within the data. Each algorithm was chosen for its unique strengths in handling binary classification problems. The LR algorithm makes predictions by modeling the probability of compound activity. The random forest algorithm combines multiple decision trees, leveraging their collective wisdom to enhance prediction accuracy. The NB algorithm, grounded in Bayes’ theorem, computes the probability of compound activity given the observed features.

The SVM algorithm maximizes the margin between different classes by identifying the optimal decision boundary. The KNN algorithm predicts the activity of new compounds based on the activity of their closest neighbors in the feature space. Finally, the MLP algorithm employs a neural network architecture, enabling the learning of complex nonlinear relationships.^37–39^ Each model was meticulously refined, maximizing its performance through hyperparameter tuning, feature selection, and the application of regularization techniques to prevent overfitting and enhance generalization capabilities.

In the present work, we selected the SVM Figure 5 model for its superior performance. SVM is a binary classifier whose basic model is defined as the linear classifier with the largest margin in feature space, and its learning strategy revolves around maximizing this margin. After applying principal component analysis (PCA) for dimensionality reduction, the features exhibited a nonlinear relationship. Hence, we opted for the Gaussian radial basis function (RBF) kernel due to its renowned performance with varying sample sizes and adaptability to diverse scenarios. Regularization was employed to enhance the stability and control the model complexity. Through fine-tuning, a validation set score of 85.71% was achieved when *C* = 5, marking an improvement of around 10% compared to the unadjusted model.

**Figure 5:**
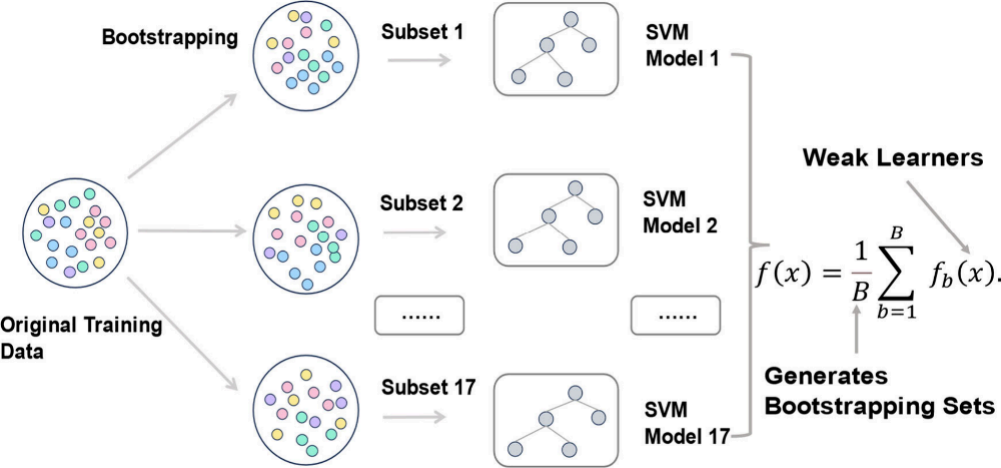
The bagging ensemble model creates 17 bootstrap samples by randomly drawing instances with replacement from the original data set. Each sample trains an SVM-based classifier, resulting in a robust ensemble learning system.

Additionally, we utilized the Bagging ensemble method to mitigate the overfitting risks. Bagging stands out for its simplicity and intuitiveness compared with other ensemble techniques like boosting. Given our small data set, the complexity of more intricate models could lead to overfitting issues, making bagging a crucial component in our approach.

We divided the data set into a training set and a test set, with 80% of the data used for training and 20% for testing. Subsequently, we further split 20% of the training data to create a validation set that was employed for model fine-tuning and hyperparameter adjustments. In terms of model comparative assessment, we employed a strategic 5-fold cross-validation approach^40^ with a strategic 5-fold approach.^41^ This method allowed us to objectively scrutinize each algorithm’s ability to generalize to new data and guard against overfitting the training data. By generating meticulous classification reports, we gained insights into the prowess of these models in correctly predicting the two classes. Furthermore, receiver operating characteristic (ROC) curves and area under the curve (AUC) values provided additional insights, enabling us to compare the strengths and weaknesses of each model and revealing their performance in terms of true positive and false-positive rates.^42^

Confusion matrices were also employed to help identify misclassified compounds and provide a means to understand potential weaknesses in the models.^43^ Through the classification reports and various evaluation metrics, we were able to comprehensively compare and select the optimal model as the primary model for further optimization. To further enhance the predictive capabilities of the model, we ventured into the realm of ensemble learning by combining the bagging method with the primary model. Given the moderate size of our data set, model complexity played a pivotal role in influencing predictive performance. The bagging technique, with its fewer parameters, reduced sensitivity to parameter selection and fostered a more adaptable understanding of data complexity.

## 3 Results and Discussion

### 3.1 Evaluation of Machine Learning Model Performance

Table 7 presents the performance of six distinct machine learning approaches in terms of accuracy, precision, recall, f1-score, and 5-fold cross-validation results. This comprehensive classification report facilitates an in-depth assessment of the models. Accuracy measures the overall ability of a model to make correct predictions, while recall focuses on its capacity to correctly identify positive instances, calculating the proportion of actual positives correctly predicted as positive. The f1-score provides a balanced assessment by combining precision and recall, making it a useful indicator of the model’s predictive power. According to Table 7, MLP, LR, SVM, and KNN demonstrate remarkable performance on both the test and validation sets. Specifically, they achieve an accuracy as high as 92.68% on the test set, indicating their strong predictive capability when presented with new, unseen data. Furthermore, these four models also exhibit impressive results in terms of precision, recall, and f1-score. To evaluate the generalizability and stability of the models, we employed 5-fold cross-validation. By comparing the cross-validation scores of MLP, LR, SVM, and KNN, it is observed that SVM exhibits slightly higher scores than the other three models. Notably, combining the performance on both test and validation sets, as illustrated in Tables 7 and 8, reveals that SVM consistently outperforms the other three algorithms.

**Table 7:** Accuracy, Precision, Recall, and f1-Scores of the Models on the Test Set, Along with Their 5-Fold Cross-Validation Scores on the Validation Set^a^

model	accuracy	precision	recall	f1-score	cross-validation score
MLP	0.9268	0.9279	0.9268	0.9268	0.7451(±0.0519)
LR	0.9268	0.9278	0.9268	0.9278	0.7138(±0.0733)
SVM	0.9268	0.9279	0.9268	0.9268	0.7659(±0.0769)
KNN	0.9268	0.9279	0.9268	0.9268	0.7298(±0.0701)
GB	0.8780	0.8788	0.8780	0.8779	0.7032(±0.0668)
NB	0.8780	0.8859	0.8780	0.8772	0.7135(±0.0578)

a Among the models, the SVM model stands out as the top performer, demonstrating superior performance across these metrics.

**Table 8:** Classification Report for the Six Models in the Validation Set^a^

model	accuracy	precision	recall	f1-score
MLP	0.7429	0.4790	0.7429	0.7403
LR	0.7714	0.7743	0.7714	0.7703
SVM	0.8000	0.8013	0.8000	0.8000
KNN	0.7714	0.8058	0.7714	0.7633
GB	0.6857	0.6868	0.6857	0.6857
NB	0.7143	0.7162	0.7143	0.7129

a The SVM model exhibits superior performance across the metrics in the table when compared to the other models.

### 3.2 Model Evaluation Based on ROC Curve and AUC

In our continued exploration of model evaluation, we turn once again to the ROC curve and AUC as valuable tools. Figure 6 displays the ROC curves for six models along with their corresponding AUC values. While subtle differences exist in the true positive rate (TPR) and false positive rate (FPR) among the models shown in Figure 6, one notable trend emerges: the TPR consistently outperforms the FPR for most models. The SVM model, which previously demonstrated high accuracy, also exhibits a favorable ROC curve and AUC value. This reinforces the model’s enhanced stability and precision in classification tasks. Furthermore, the ROC curve provides a visual representation of the model’s performance at various threshold settings, while the AUC value solidifies its reputation as a robust classifier.

**Figure 6:**
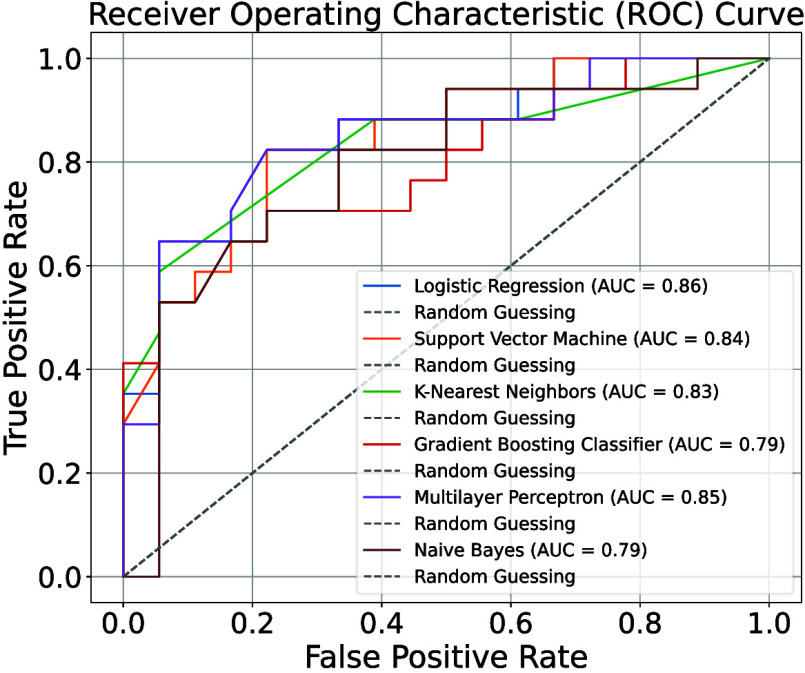
After comparing the AUC curves of six different models, with consistent input values, training sets, and validation sets, the logistic regression (LR), support vector machine (SVM), and multilayer perceptron (MLP) models stood out for their ability to correctly identify positive instances.

### 3.3 Confusion Matrix Analysis

Building upon the initial insights gained from the classification reports, ROC curves, and AUC values, we identified the SVM as a standout model, particularly excelling in accuracy and generalizability. To further substantiate the capabilities of these models and visualize their performance data, we turn to confusion matrices, which offer a subtle summary of the interplay between the model predictions and the test set. Confusion matrices, with their tabular elegance, present a synthesis of true values and model predictions, unveiling the models’ performance across categories and shedding light on any potential class imbalance issues. As illustrated in Figure 7a–f, the SVM model consistently demonstrates remarkable performance in predicting compound activity. These confusion matrices corroborate the robust performance of the SVM model, once again underscoring its prowess in tackling binary classification challenges. They provide a clear depiction of the true and false positives and negatives, enabling an assessment of the models’ accuracy, precision, and recall.

**Figure 7:**
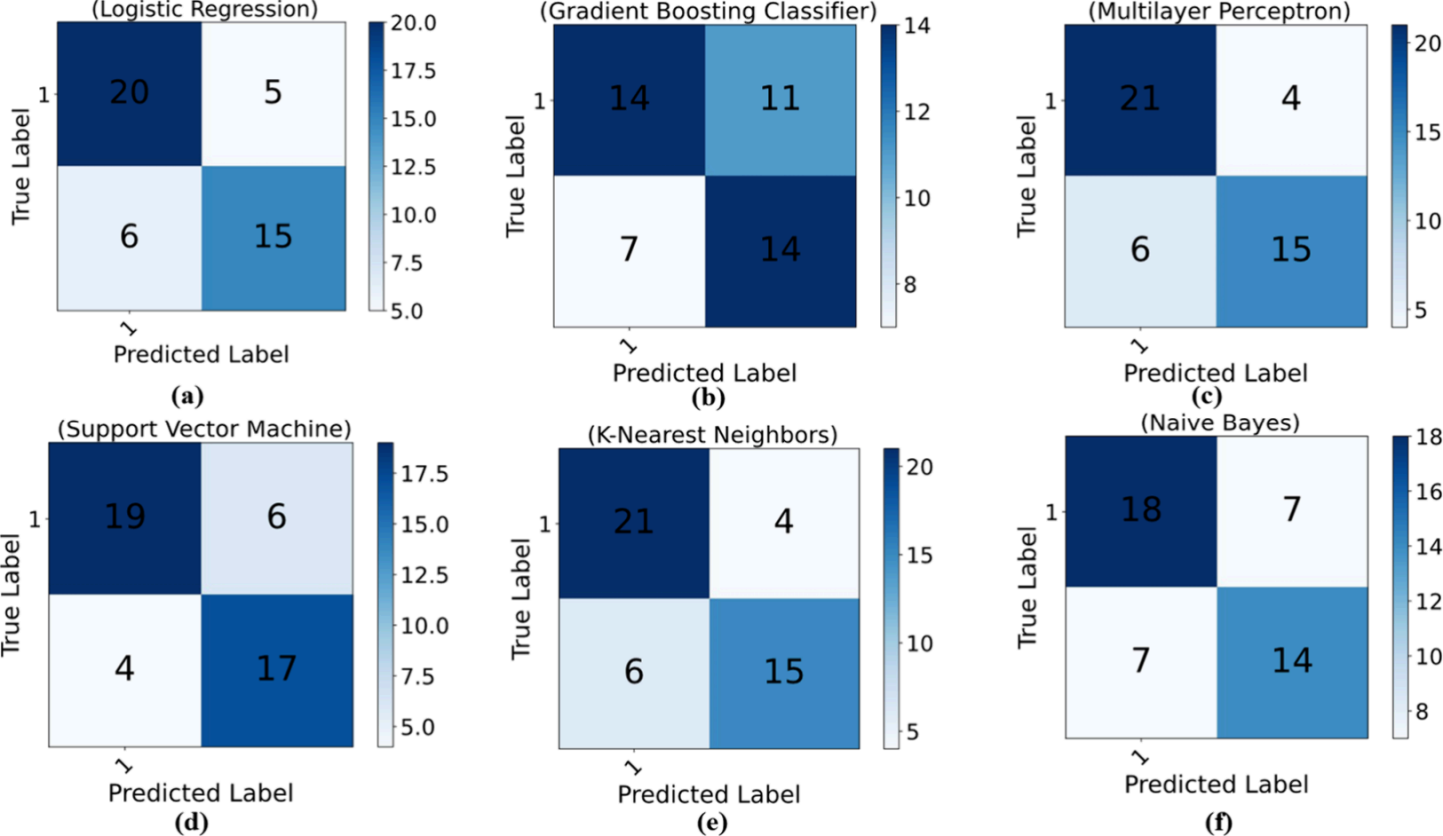
With identical input values of *X* and target variable activity (*Y*), along with consistent training and validation sets, we compared the confusion matrices of six distinct models. Among them, the K-nearest neighbors (KNN) and multilayer perceptron (MLP) models, alongside the support vector machine (SVM) model, emerged as the top performers.

### 3.4 Learning Curve Analysis and Model Optimization

To unravel the intricacies of the SVM model’s performance and detect any potential overfitting or underfitting issues, we turn to learning curves, elegantly depicted in Figure 8. Through a meticulous analysis of the results illustrated in this figure, we observe that the SVM model exhibits a small gap between the training score and cross-validation score curves, indicating the absence of overfitting. Additionally, the high scores attained by the SVM model reinforce its strength as a viable choice.

**Figure 8:**
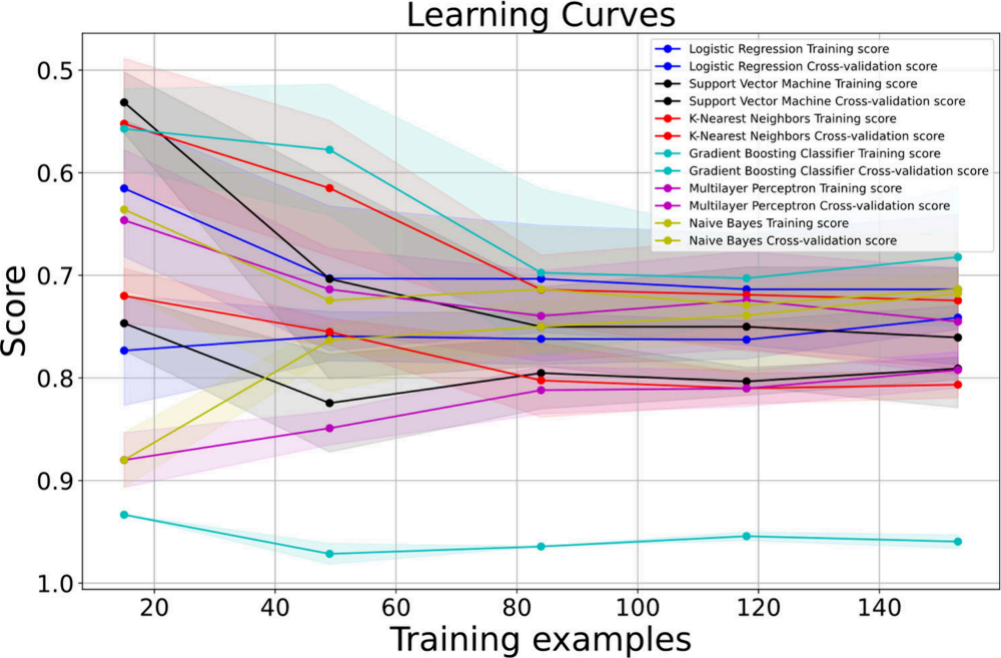
SVM model shows excellent performance, with training scores close to cross-validation scores, demonstrating strong generalization ability and no apparent overfitting.

After a rigorous selection process, the SVM emerged as the top-performing model and was chosen for further optimization. To enhance the SVM model, we employed an ensemble approach based on SVM classifiers: bagging. By fine-tuning the parameters and referencing the validation set scores, the integrated model achieved an average improvement of approximately 3% in precision, recall, and f1-score (as illustrated in Figure 9). Remarkably, it showed an increase of 10% in the validation set. The ROC curve shifted closer to the top-left corner, and the AUC value improved from 84% to 95%. These outcomes validate the effectiveness of our chosen ensemble method and demonstrate how the Bagging integration enhances the model’s stability and generalization capabilities, leading to superior performance.

**Figure 9:**
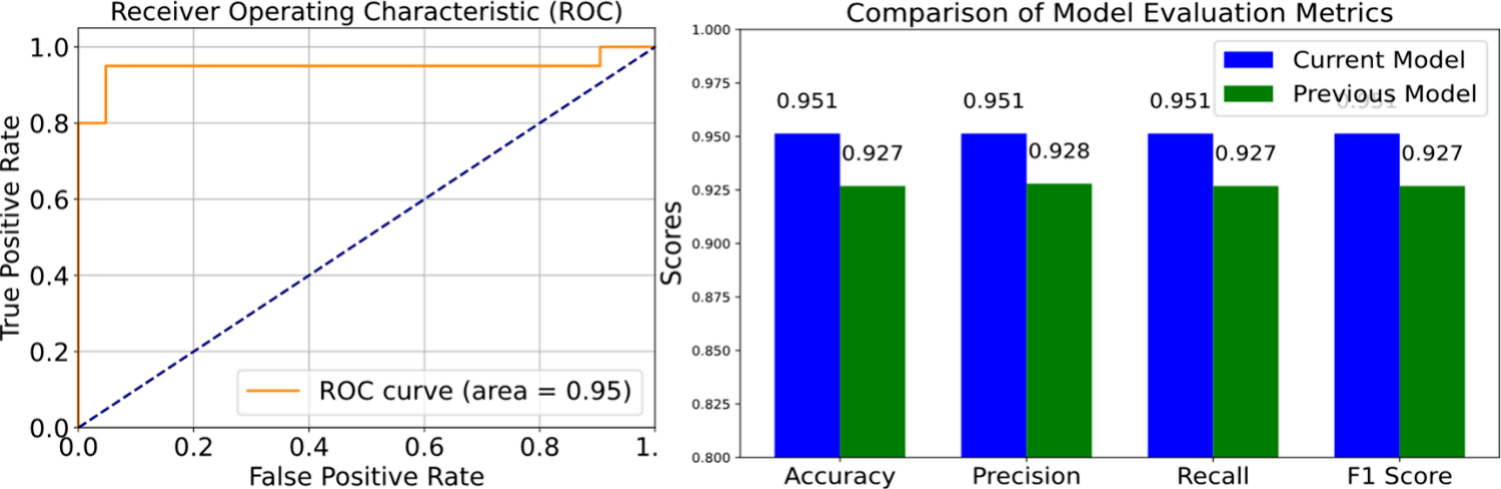
The optimized model exhibits enhanced performance, evident by the ROC curve shifting toward the upper left corner and an increased AUC value of 95%. Improvements are also reflected in the precision, recall, and f1-score, each showing an approximately 3% increase. These advancements signify the model’s improved capability to retain positive instances while reducing misclassified negative cases.

### 3.5 Analysis of Model Prediction Results and Exploration of Chemical Mechanisms

Figure 10 illustrates the comparison between the predicted probabilities and the actual outcomes of the molecules. According to the graph, the model accurately predicted the categories of most molecules, but the probability distribution is unstable, possibly due to the small data set leading to insufficient learning. Nonetheless, the model’s performance is considered satisfactory for classification tasks. Based on the results presented in Figure 10, of 41 unknown compounds, only 2 were misidentified, resulting in an error rate of approximately 4.8%. This model’s classification capability is suitable for practical classification tasks.

**Figure 10:**
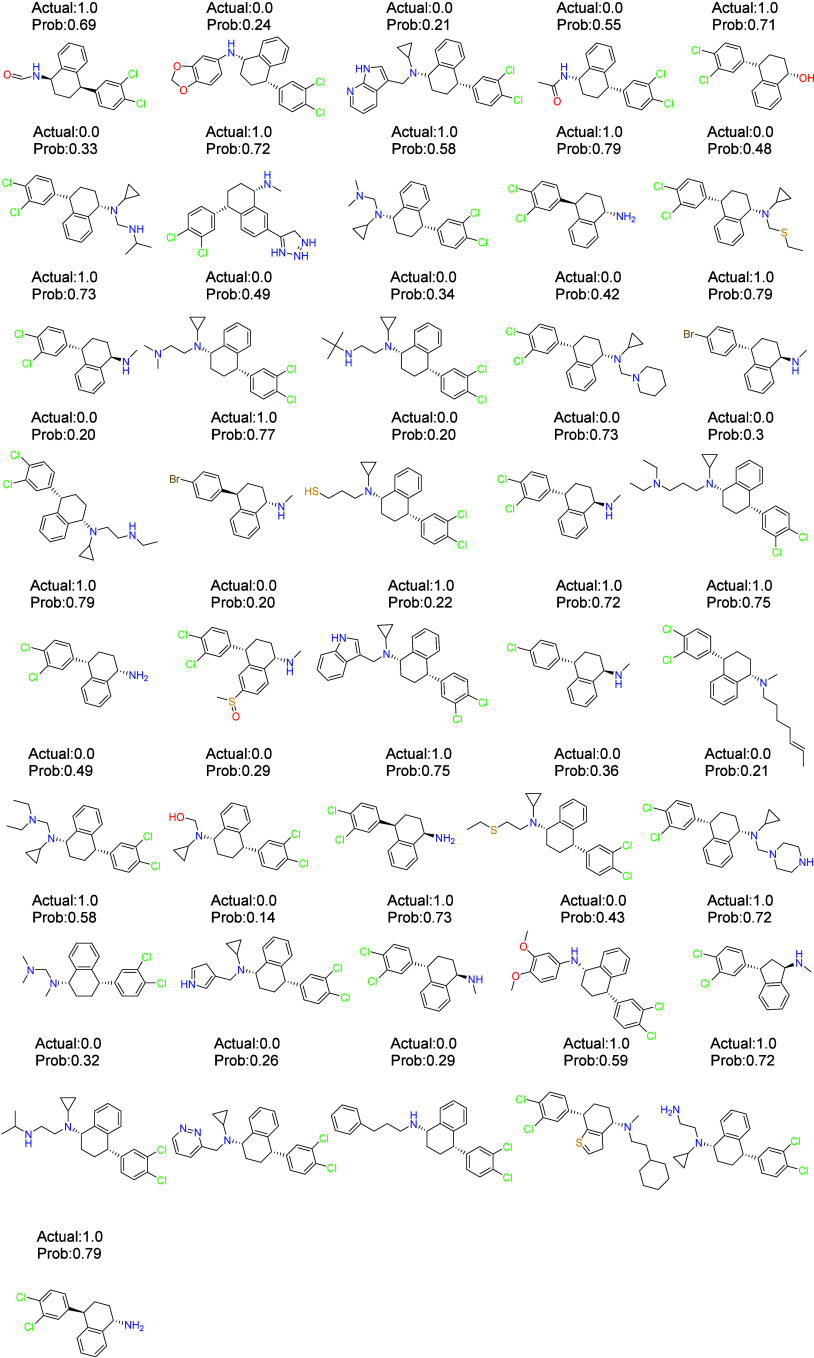
Actual values and predicted probability values of the molecules according to the model.

Analysis of the model’s predicted compound results revealed that the presence of H on secondary amines was explicitly captured during training, showing a strong correlation with compound activity. In the test set (Figure 9), 12 compounds with N–H on secondary amines and actual activity were identified, all of which received model predictions above 0.5, with a confidence level of 100%. This strongly indicates that the model successfully established a positive association between amine H and activity based on the training data. From a chemical mechanism perspective, the unpaired electron on the amine H may participate in target binding through hydrogen bonding or electrostatic interactions, thereby enhancing compound activity.

However, some compounds with N–H on secondary amines exhibited no activity. Upon analysis, it appears that bulky substituents on the secondary amines may significantly hinder the conformational fitting required for target binding. As shown in Figure 11, despite the presence of N–H on the secondary amines in these compounds, the large substituents introduce steric hindrance, making it difficult to form hydrogen bonds.^25^

**Figure 11:**
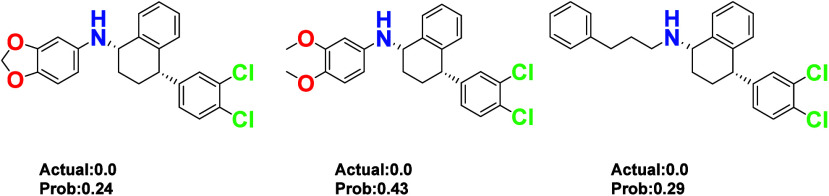
Compounds with N–H on secondary amines show neither actual nor predicted activity.

In the model’s prediction results, it was also observed that compounds remained active when the chlorine substituent on the benzene ring was replaced with bromine or when the ortho-chlorine was removed (Figure 12). The model accurately identified compounds containing chlorine (Cl) or bromine (Br) on the benzene ring as active (predicted value of >0.5). According to literature,^26^ the hydrophobic effect of dichlorophenyl groups can enhance molecular binding to the target’s hydrophobic cavity. Bromine, with its larger atomic radius, may exert a stronger hydrophobic effect, while chlorine and bromine exhibit similar electronic effects (σ_p ≈ 0.23 vs 0.26). Therefore, the model did not misjudge due to changes in the halogen type.

**Figure 12:**
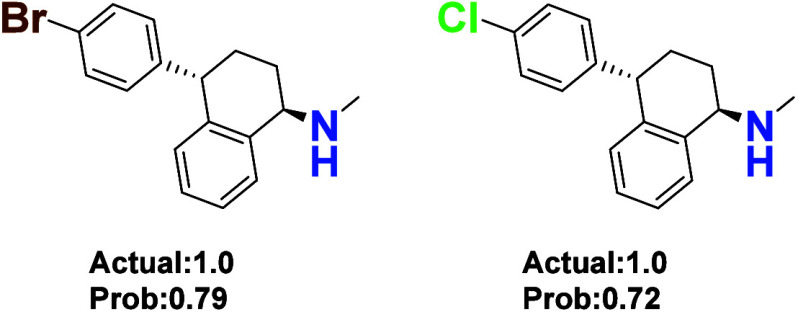
Compounds with chlorine substituents on the benzene ring replaced by bromine or with ortho-position chlorine removed still exhibit activity.

The model has captured key structural features that provide important guidance for the design of candidate compounds. The model’s precise identification of N–H on secondary amines as a critical marker of activity suggests that N–H should be retained during molecular modification to prevent activity loss. Additionally, the model’s ability to recognize the activity of bromine-substituted compounds among numerous dichlorophenyl-containing compounds demonstrates not only its understanding of small molecule structural relationships but also its accurate capture of hydrophobic interaction features at this position. This may be attributed to the model’s effective learning of small molecule properties such as log *P*, log *S*, and topological polar surface area (TPSA). Based on these findings, in small molecule modification predictions, exploring the replacement of chlorine substituents on dichlorophenyl groups with other halogen atoms is a promising strategy.

## 4 Conclusion

In summary, our chosen SVM model, enhanced with the bagging integration method, excels in both predictive and generalization capabilities. Its strong predictive performance indicates the model’s potential for practical development of sertraline analogs, streamlining the development cycle and reducing costs associated with synthesizing inactive compounds. Additionally, the model’s exceptional generalization ability extends to predicting similar compounds, offering valuable insights into compound data sets and activity data. By leveraging this analytical framework, we can efficiently predict the activity of related compounds, expediting the experimental process.

## Data Availability

All data and code presented in this study can be found at https://github.com/xiaxiazainuli/A-Machine-Learning-Model-for-Predicting-Sertraline-like-Activities.git, where the data and code can be accessed through the Web site.
